# Supplementary figures and images for: Epidemiological Transitions in Influenza Dynamics in the United States: Insights from Recent Pandemic Challenges
Source: Microorganisms. 2025 Feb 20;13(3):469. doi: 10.3390/microorganisms13030469 (PMC11945264; doi:10.3390/microorganisms13030469)

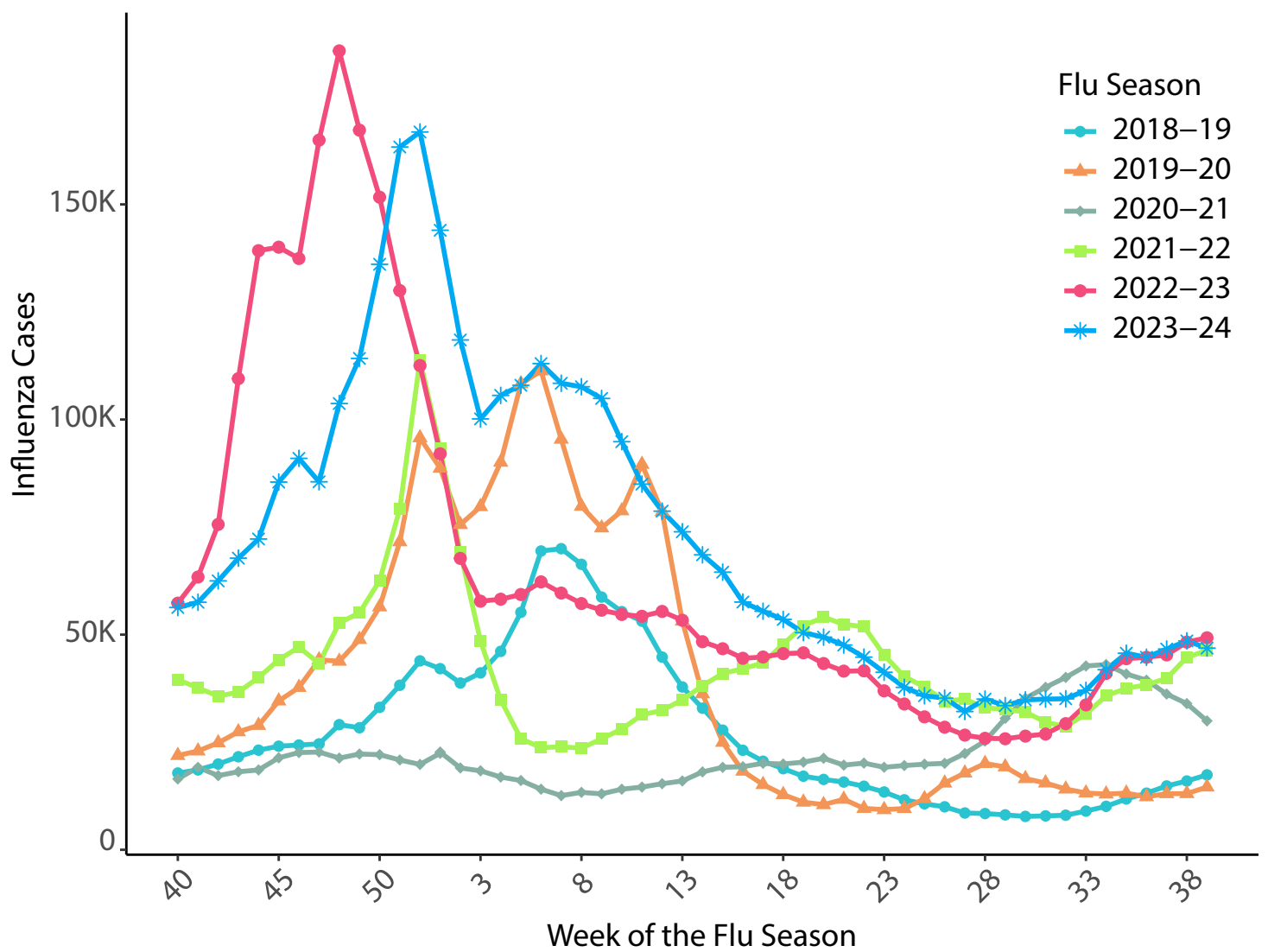

Supplement: Supplementary file 1 [file microorganisms-13-00469-s001.zip › Supplementary Figure S1.pdf]

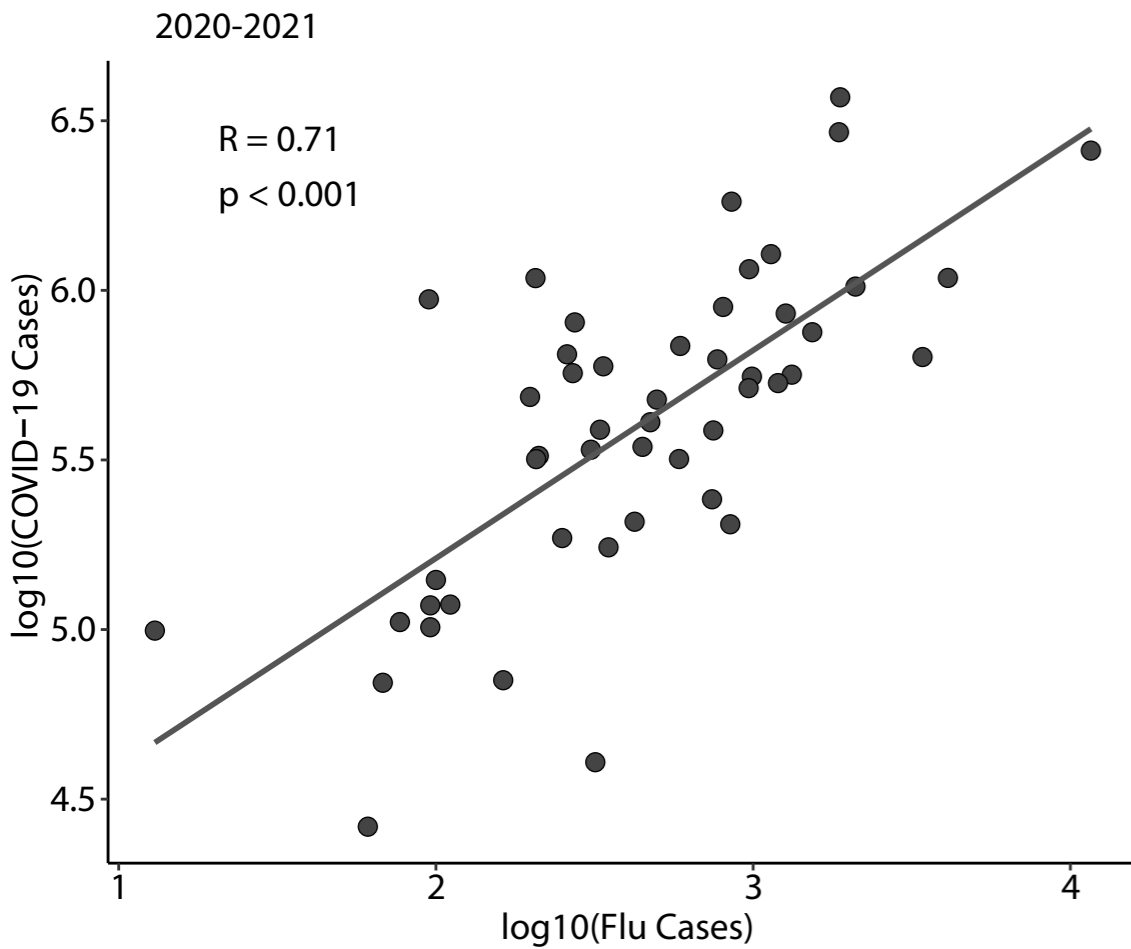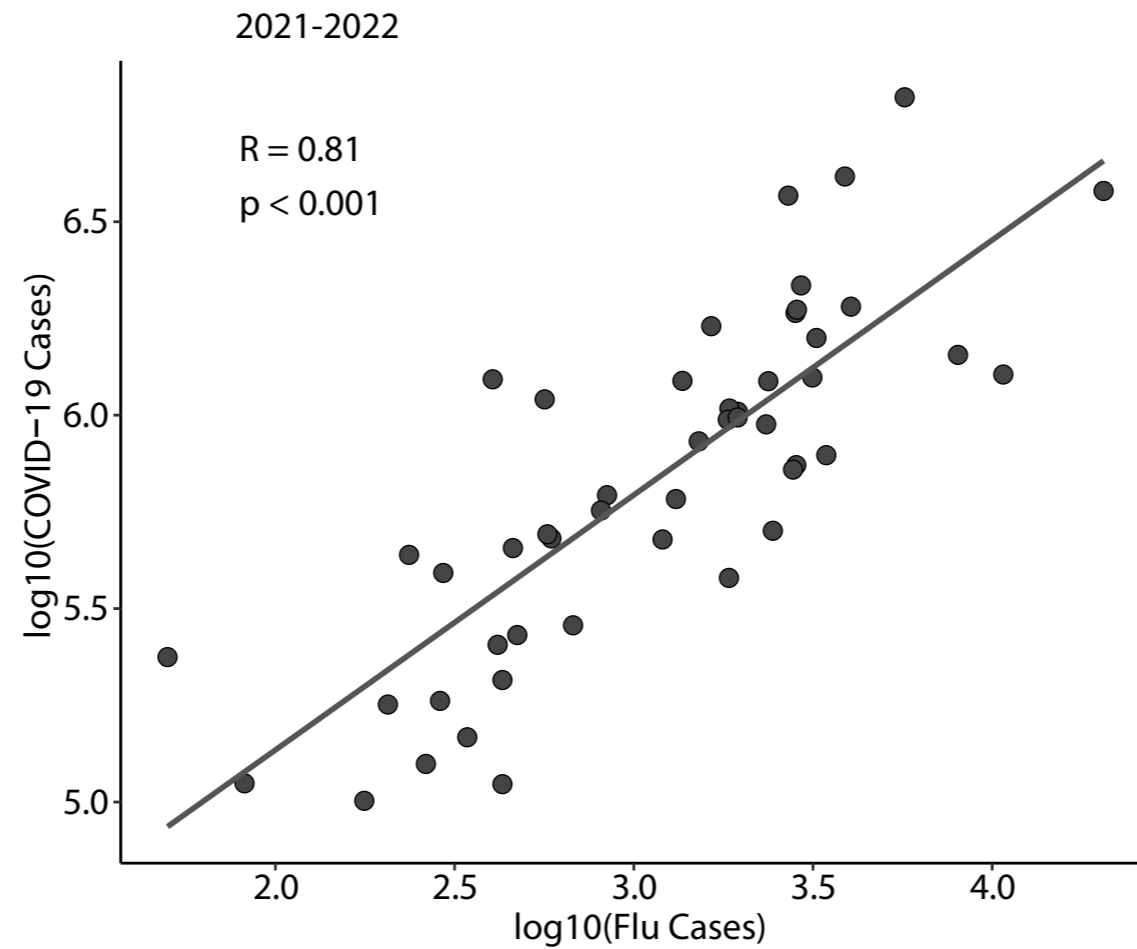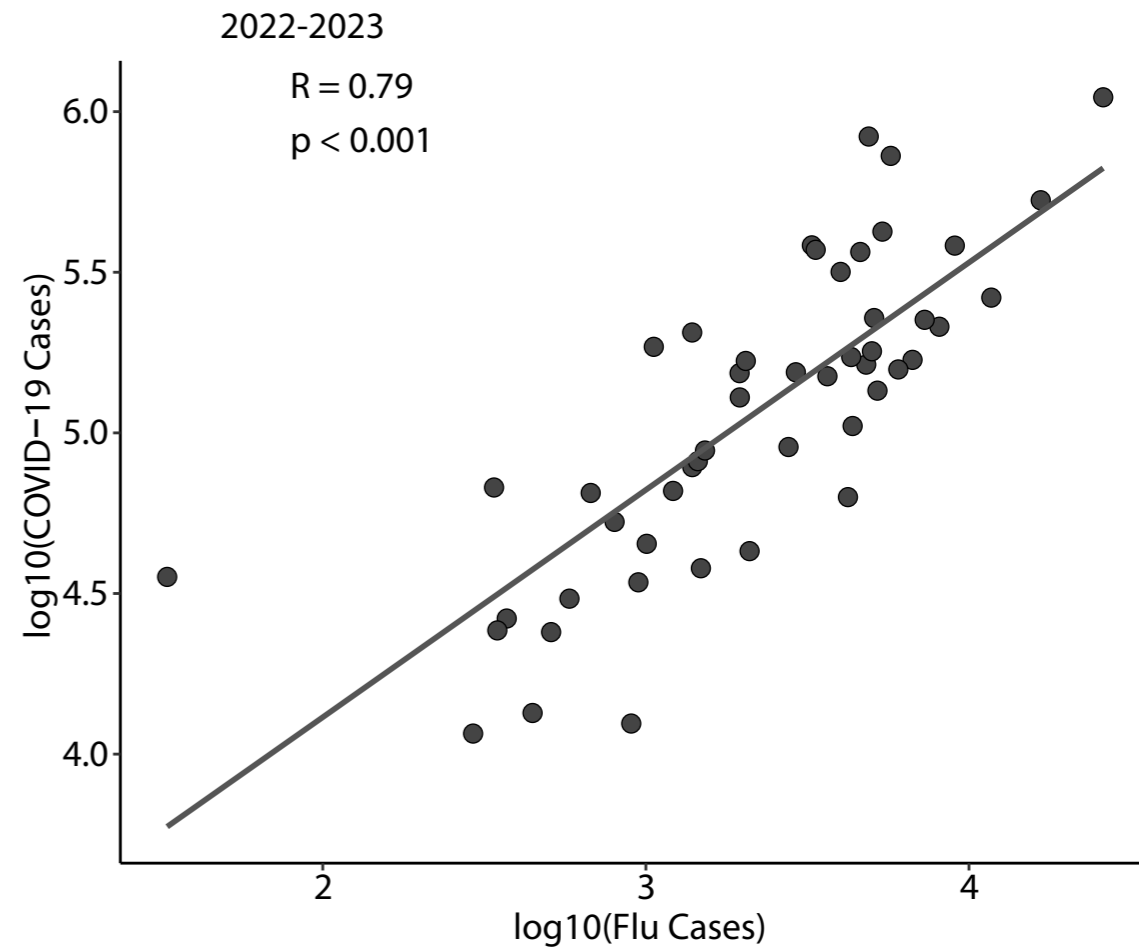

Supplement: Supplementary file 1 [file microorganisms-13-00469-s001.zip › Supplementary Figure S2.pdf]

**a**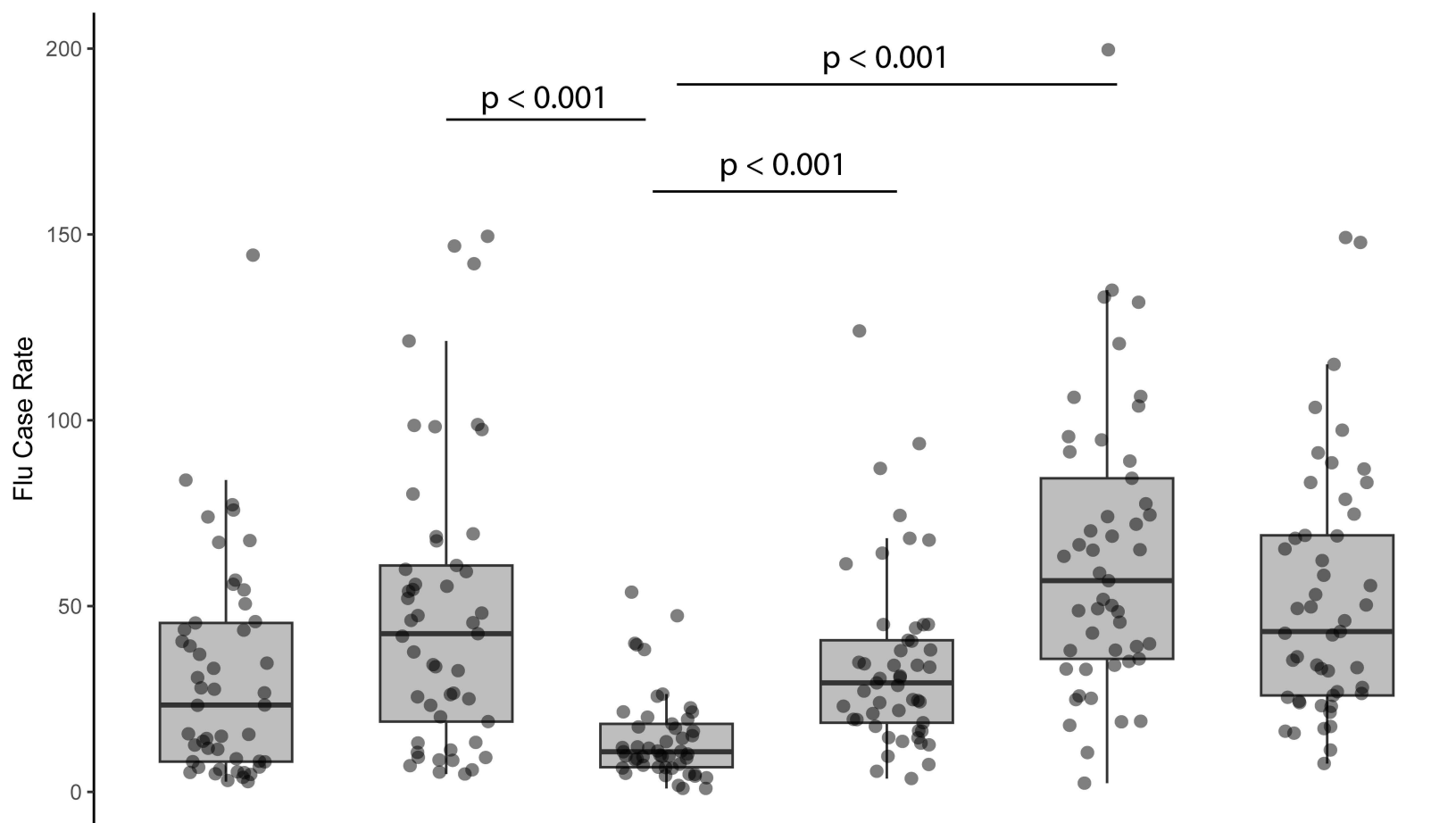**b**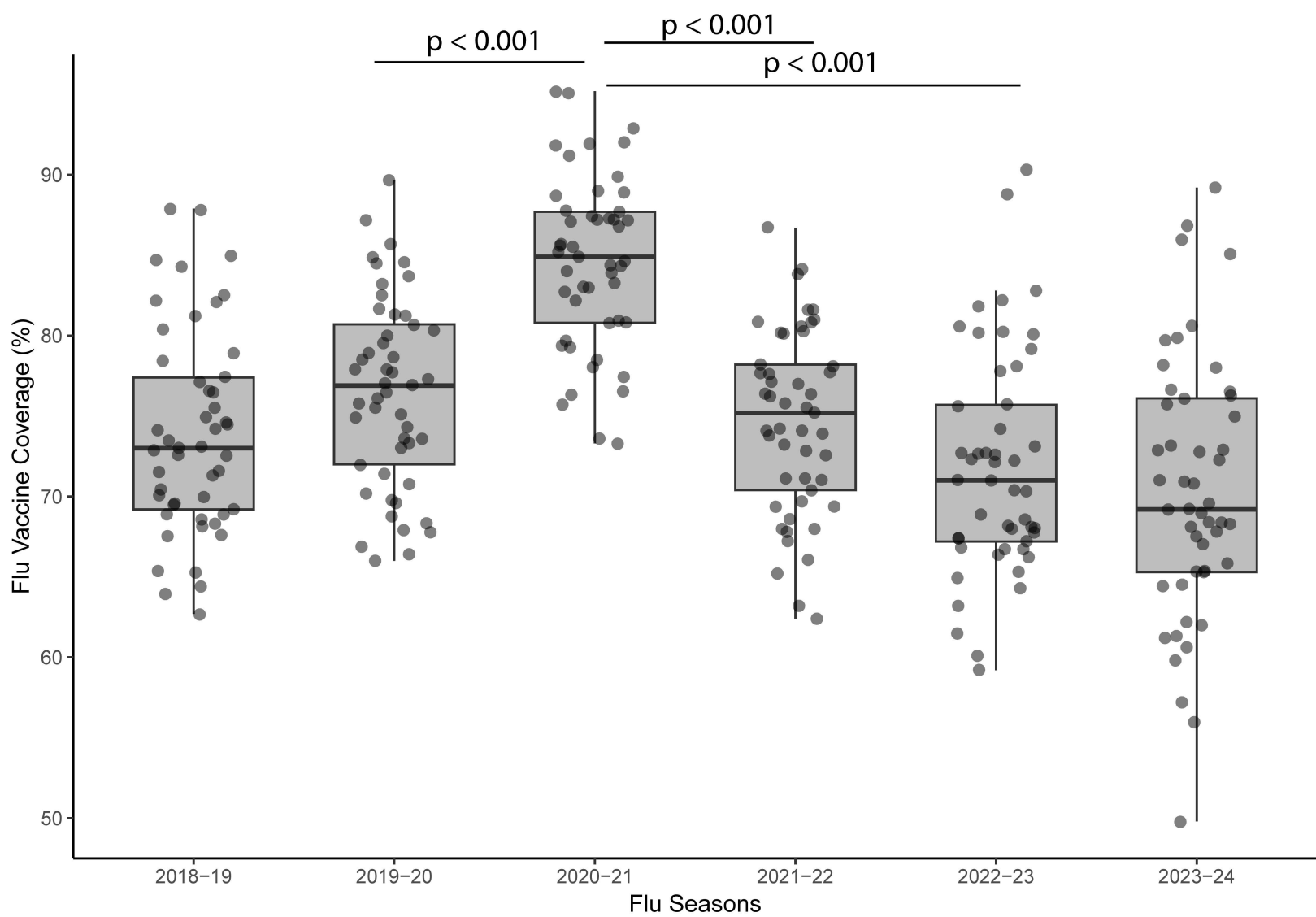

Supplement: Supplementary file 1 [file microorganisms-13-00469-s001.zip › Supplementary Figure S3_revised.pdf]
